# Bis(2-{5-[(2-carb­oxy­phen­yl)sulfanylmeth­yl]-2,4-dimethyl­benzyl­sulfan­yl}benzoato-κ^2^ *O*,*O*′)bis­(pyridine-κ*N*)iron(II)

**DOI:** 10.1107/S1600536810054619

**Published:** 2011-01-12

**Authors:** Yu-Min Xu, Tuo-Ping Hu

**Affiliations:** a Department of Chemistry, North University of China, Taiyuan 030051, People’s Republic of China

## Abstract

The title compound, [Fe(C_24_H_21_O_4_S_2_)_2_(C_5_H_5_N)_2_], has 2 symmetry. The Fe^II^ cation is located on a twofold rotation axis and is *O*,*O*′-chelated by two 2-{5-[(2-carb­oxy­phen­yl)sulfanylmeth­yl]-2,4-dimethyl­benzyl­sulfan­yl}benzoate anions and further coordinated by two pyridine ligands in a distorted octa­hedral geometry. In the anion, the terminal benzene rings are oriented at dihedral angles of 63.81 (14) and 84.50 (14)° with respect to the central benzene ring. Inter­molecular O—H⋯O and C—H⋯O hydrogen bonding is present in the crystal structure.

## Related literature

For applications of multithio­ether ligands in inorganic chemistry, see: Li *et al.* (2002 ▶). For structures of related complexes with multithio­ether ligands, see: Bu *et al.* (2002 ▶); Alcock *et al.* (1978 ▶) and with carboxylate ligands, see: Dai *et al.* (2008 ▶).
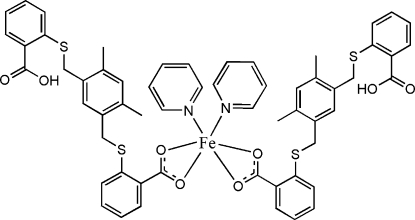


## Experimental

### 

#### Crystal data

[Fe(C_24_H_21_O_4_S_2_)_2_(C_5_H_5_N)_2_]
*M* *_r_* = 1089.10
Orthorhombic, 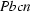

*a* = 16.987 (5) Å
*b* = 9.635 (3) Å
*c* = 31.982 (10) Å
*V* = 5234 (3) Å^3^
*Z* = 4
Mo *K*α radiation
μ = 0.51 mm^−1^
*T* = 293 K
0.15 × 0.10 × 0.10 mm

#### Data collection

Bruker APEXII CCD area-detector diffractometer
Absorption correction: multi-scan (*SADABS*; Sheldrick, 1996 ▶) *T*_min_ = 0.928, *T*_max_ = 0.951
29090 measured reflections
5950 independent reflections
3431 reflections with *I* > 2σ(*I*)
*R*_int_ = 0.073

#### Refinement

*R*[*F* ^2^ > 2σ(*F* ^2^)] = 0.047
*wR*(*F* ^2^) = 0.114
*S* = 1.00
5950 reflections
336 parameters
H atoms treated by a mixture of independent and constrained refinement
Δρ_max_ = 0.29 e Å^−3^
Δρ_min_ = −0.24 e Å^−3^

### 

Data collection: *APEX2* (Bruker, 2007 ▶); cell refinement: *SAINT-Plus* (Bruker, 2007 ▶); data reduction: *SAINT-Plus*; program(s) used to solve structure: *SHELXTL* (Sheldrick, 2008 ▶); program(s) used to refine structure: *SHELXTL*; molecular graphics: *SHELXTL*; software used to prepare material for publication: *SHELXTL*.

## Supplementary Material

Crystal structure: contains datablocks global, I. DOI: 10.1107/S1600536810054619/xu5127sup1.cif

Structure factors: contains datablocks I. DOI: 10.1107/S1600536810054619/xu5127Isup2.hkl

Additional supplementary materials:  crystallographic information; 3D view; checkCIF report

## Figures and Tables

**Table 1:** Selected bond lengths (Å)

Fe1—O1	2.412 (2)
Fe1—O2	2.0400 (18)
Fe1—N1	2.100 (2)

**Table 2:** Hydrogen-bond geometry (Å, °)

*D*—H⋯*A*	*D*—H	H⋯*A*	*D*⋯*A*	*D*—H⋯*A*
O4—H4⋯O1^i^	0.96 (4)	1.77 (4)	2.702 (3)	162 (4)
C16—H16*A*⋯O3^ii^	0.93	2.54	3.421 (3)	158

Symmetry codes: (i) 
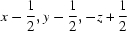
; (ii) 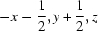.

**Fig. 1.**
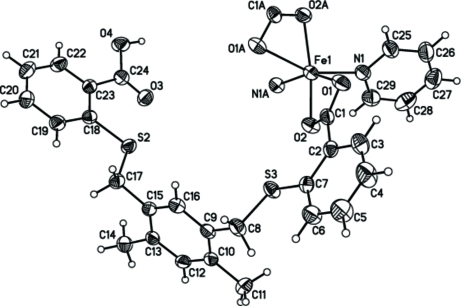
Molecular structure showing 35% probability displacement ellipsoids. The atoms marked with A are derived from the reference atoms by the symmetry transformation of (-x, y, z).

## supplementary crystallographic information

### Comment

Multithioether ligands possess unusual potential for structure control in inorganic chemistry (Li et al., 2002). Some crystal structures of complexes with multithioether ligands have been reported previously (Bu et al., 2002; Alcock et al., 1978). The title compound with the flexible multithioether ligand was obtained in preparing of metal-organic framework (MOF) compounds.

In (I), the central Fe^2+^ ion is coordinated by four oxygen atoms from the carboxylate group of the ligand and two nitrogen atoms from the pyridine molecule in a distorted octahedron (Fig. 1).

The asymmetry unit contains one ligand, one pyridine molecule, and half Fe^2+^ ion sitting on the twofold axis, with the other part being generated by the symmetry operation of C<ι>_2_.

Strong H-bondings are observed between the O-H group of the carboxylate group and O2 in the  crystal structure (Table 2).

### Experimental

2,4-Bis(2-(benzylacid)thiophenylmethyl)-1,5-dimethylbenze (2 mg, 4.6 mmol) and FeSO_4_ (4 mg, 26.3 mmol) were dissolved in 1 ml mixed solution of H2O, DMF and MeOH (5:3:2), then 1 drop pyridine was added. The mixed solution was sealed into a Pyrex glass tube. The mixed solution was heated at 363 K for 60 h, and then cooled down to room temperature over 17 h. Orange crystals were obtained from the reaction mixture.

### Refinement

The carboxyl H atom was located in a difference Fourier map and refined freely. Other H atoms were placed in calculated positions with C—H = 0.93–0.97 Å and refined in riding mode, *U*_iso_(H) = 1.2*U*_eq_(C).

### Crystal data

**Table:**

[Fe(C_24_H_21_O_4_S_2_)_2_(C_5_H_5_N)_2_]	*F*(000) = 2272
*M**_r_* = 1089.10	*D*_x_ = 1.382 Mg m^−^^3^
Orthorhombic, *P**b**c**n*	Mo *K*α radiation, λ = 0.71073 Å
Hall symbol: -P 2n 2ab	Cell parameters from 3184 reflections
*a* = 16.987 (5) Å	θ = 2.4–20.3°
*b* = 9.635 (3) Å	µ = 0.51 mm^−^^1^
*c* = 31.982 (10) Å	*T* = 293 K
*V* = 5234 (3) Å^3^	Prism, orange
*Z* = 4	0.15 × 0.10 × 0.10 mm

### Data collection

**Table:**

Bruker APEXII CCD area-detector diffractometer	5950 independent reflections
Radiation source: fine-focus sealed tube	3431 reflections with *I* > 2σ(*I*)
graphite	*R*_int_ = 0.073
φ and ω scans	θ_max_ = 27.4°, θ_min_ = 1.8°
Absorption correction: multi-scan (*SADABS*; Sheldrick, 1996)	*h* = −22→18
*T*_min_ = 0.928, *T*_max_ = 0.951	*k* = −11→12
29090 measured reflections	*l* = −40→41

### Refinement

**Table:**

Refinement on *F*^2^	Primary atom site location: structure-invariant direct methods
Least-squares matrix: full	Secondary atom site location: difference Fourier map
*R*[*F*^2^ > 2σ(*F*^2^)] = 0.047	Hydrogen site location: inferred from neighbouring sites
*wR*(*F*^2^) = 0.114	H atoms treated by a mixture of independent and constrained refinement
*S* = 1.00	*w* = 1/[σ^2^(*F*_o_^2^) + (0.0476*P*)^2^ + 0.3652*P*] where *P* = (*F*_o_^2^ + 2*F*_c_^2^)/3
5950 reflections	(Δ/σ)_max_ < 0.001
336 parameters	Δρ_max_ = 0.29 e Å^−^^3^
0 restraints	Δρ_min_ = −0.24 e Å^−^^3^

### Special details

**Table:**

Geometry. All e.s.d.'s (except the e.s.d. in the dihedral angle between two l.s. planes) are estimated using the full covariance matrix. The cell e.s.d.'s are taken into account individually in the estimation of e.s.d.'s in distances, angles and torsion angles; correlations between e.s.d.'s in cell parameters are only used when they are defined by crystal symmetry. An approximate (isotropic) treatment of cell e.s.d.'s is used for estimating e.s.d.'s involving l.s. planes.
Refinement. Refinement of *F*^2^ against ALL reflections. The weighted *R*-factor *wR* and goodness of fit *S* are based on *F*^2^, conventional *R*-factors *R* are based on *F*, with *F* set to zero for negative *F*^2^. The threshold expression of *F*^2^ > σ(*F*^2^) is used only for calculating *R*-factors(gt) *etc*. and is not relevant to the choice of reflections for refinement. *R*-factors based on *F*^2^ are statistically about twice as large as those based on *F*, and *R*- factors based on ALL data will be even larger.

### Fractional atomic coordinates and isotropic or equivalent isotropic displacement parameters (Å^2^)

**Table:**

	*x*	*y*	*z*	*U*_iso_*/*U*_eq_	
Fe1	0.0000	0.74627 (6)	0.2500	0.04331 (16)	
S2	−0.30599 (4)	0.78127 (7)	0.40440 (2)	0.04618 (19)	
S3	0.04300 (4)	0.89147 (7)	0.38414 (2)	0.0475 (2)	
N1	0.08272 (13)	0.6062 (2)	0.22545 (7)	0.0479 (6)	
O1	0.09558 (11)	0.9313 (2)	0.25198 (6)	0.0540 (5)	
O2	0.05286 (11)	0.8060 (2)	0.30437 (5)	0.0499 (5)	
O3	−0.35938 (12)	0.5896 (2)	0.34618 (6)	0.0610 (6)	
O4	−0.45225 (12)	0.6383 (2)	0.29905 (6)	0.0617 (6)	
C1	0.09241 (15)	0.9076 (3)	0.29042 (8)	0.0417 (6)	
C2	0.13707 (15)	0.9951 (3)	0.32090 (8)	0.0396 (6)	
C3	0.19723 (17)	1.0779 (3)	0.30537 (10)	0.0575 (8)	
H3A	0.2079	1.0777	0.2768	0.069*	
C4	0.2413 (2)	1.1602 (4)	0.33156 (11)	0.0749 (10)	
H4A	0.2820	1.2143	0.3209	0.090*	
C5	0.22487 (19)	1.1615 (4)	0.37331 (11)	0.0755 (10)	
H5A	0.2546	1.2171	0.3911	0.091*	
C6	0.16505 (17)	1.0818 (3)	0.38954 (10)	0.0582 (8)	
H6A	0.1545	1.0856	0.4180	0.070*	
C7	0.12002 (14)	0.9957 (3)	0.36411 (8)	0.0401 (6)	
C8	0.02048 (16)	0.9718 (3)	0.43415 (8)	0.0519 (8)	
H8A	0.0658	0.9674	0.4525	0.062*	
H8B	0.0062	1.0684	0.4302	0.062*	
C9	−0.04780 (15)	0.8916 (3)	0.45280 (8)	0.0409 (6)	
C10	−0.03712 (15)	0.7868 (3)	0.48216 (8)	0.0426 (6)	
C11	0.04427 (16)	0.7410 (3)	0.49569 (10)	0.0642 (9)	
H11A	0.0403	0.6564	0.5114	0.096*	
H11B	0.0676	0.8118	0.5128	0.096*	
H11C	0.0764	0.7259	0.4714	0.096*	
C12	−0.10259 (15)	0.7253 (3)	0.49995 (8)	0.0437 (7)	
H12A	−0.0950	0.6574	0.5202	0.052*	
C13	−0.17944 (14)	0.7601 (3)	0.48894 (8)	0.0400 (6)	
C14	−0.24754 (16)	0.6930 (3)	0.51124 (9)	0.0556 (8)	
H14A	−0.2286	0.6178	0.5282	0.083*	
H14B	−0.2844	0.6580	0.4911	0.083*	
H14C	−0.2731	0.7603	0.5287	0.083*	
C15	−0.19034 (15)	0.8607 (3)	0.45766 (8)	0.0394 (6)	
C16	−0.12421 (15)	0.9235 (3)	0.44033 (8)	0.0436 (7)	
H16A	−0.1314	0.9896	0.4195	0.052*	
C17	−0.27102 (14)	0.9053 (3)	0.44325 (8)	0.0452 (7)	
H17A	−0.2684	0.9975	0.4311	0.054*	
H17B	−0.3069	0.9080	0.4668	0.054*	
C18	−0.40055 (15)	0.8453 (3)	0.39068 (8)	0.0393 (6)	
C19	−0.43241 (17)	0.9630 (3)	0.40961 (9)	0.0493 (7)	
H19A	−0.4036	1.0091	0.4301	0.059*	
C20	−0.50547 (18)	1.0123 (3)	0.39868 (9)	0.0584 (8)	
H20A	−0.5250	1.0916	0.4116	0.070*	
C21	−0.55010 (18)	0.9458 (4)	0.36882 (10)	0.0642 (9)	
H21A	−0.6002	0.9779	0.3622	0.077*	
C22	−0.51971 (17)	0.8319 (3)	0.34908 (9)	0.0535 (8)	
H22A	−0.5494	0.7876	0.3286	0.064*	
C23	−0.44520 (15)	0.7808 (3)	0.35894 (8)	0.0400 (6)	
C24	−0.41396 (17)	0.6604 (3)	0.33493 (9)	0.0453 (7)	
C25	0.12280 (17)	0.6396 (3)	0.19087 (9)	0.0557 (8)	
H25A	0.1064	0.7160	0.1754	0.067*	
C26	0.18633 (19)	0.5669 (4)	0.17738 (11)	0.0692 (9)	
H26A	0.2131	0.5947	0.1534	0.083*	
C27	0.2106 (2)	0.4539 (4)	0.19880 (13)	0.0750 (10)	
H27A	0.2545	0.4041	0.1901	0.090*	
C28	0.1694 (2)	0.4137 (4)	0.23362 (13)	0.0778 (11)	
H28A	0.1840	0.3350	0.2486	0.093*	
C29	0.1056 (2)	0.4935 (4)	0.24592 (10)	0.0650 (9)	
H29A	0.0776	0.4668	0.2696	0.078*	
H4	−0.428 (2)	0.561 (4)	0.2852 (12)	0.124 (16)*	

### Atomic displacement parameters (Å^2^)

**Table:**

	*U*^11^	*U*^22^	*U*^33^	*U*^12^	*U*^13^	*U*^23^
Fe1	0.0466 (3)	0.0494 (3)	0.0340 (3)	0.000	0.0038 (3)	0.000
S2	0.0433 (4)	0.0469 (4)	0.0484 (4)	0.0056 (3)	−0.0105 (3)	−0.0091 (3)
S3	0.0501 (4)	0.0531 (4)	0.0393 (4)	−0.0149 (4)	0.0100 (3)	−0.0041 (3)
N1	0.0503 (14)	0.0503 (14)	0.0429 (14)	0.0007 (12)	0.0031 (11)	0.0009 (12)
O1	0.0674 (13)	0.0589 (13)	0.0357 (11)	0.0015 (10)	0.0065 (10)	0.0067 (10)
O2	0.0601 (13)	0.0514 (12)	0.0382 (11)	−0.0130 (10)	0.0019 (9)	0.0003 (9)
O3	0.0561 (13)	0.0642 (13)	0.0627 (14)	0.0133 (11)	−0.0122 (11)	−0.0187 (11)
O4	0.0714 (15)	0.0633 (14)	0.0503 (13)	0.0041 (12)	−0.0190 (11)	−0.0164 (11)
C1	0.0415 (16)	0.0433 (16)	0.0403 (17)	0.0086 (13)	0.0076 (13)	0.0033 (13)
C2	0.0367 (15)	0.0372 (15)	0.0447 (16)	0.0035 (12)	0.0043 (12)	0.0057 (12)
C3	0.0578 (19)	0.0573 (19)	0.0572 (19)	−0.0092 (16)	0.0151 (16)	0.0032 (15)
C4	0.066 (2)	0.080 (2)	0.079 (3)	−0.0352 (19)	0.021 (2)	0.000 (2)
C5	0.064 (2)	0.089 (3)	0.074 (2)	−0.038 (2)	0.0041 (18)	−0.015 (2)
C6	0.0551 (19)	0.069 (2)	0.0500 (18)	−0.0194 (16)	0.0049 (15)	−0.0061 (16)
C7	0.0343 (15)	0.0415 (15)	0.0446 (16)	−0.0018 (12)	0.0030 (12)	−0.0001 (12)
C8	0.0484 (18)	0.0641 (19)	0.0432 (17)	−0.0151 (15)	0.0091 (13)	−0.0108 (15)
C9	0.0411 (16)	0.0500 (16)	0.0315 (14)	−0.0089 (13)	0.0049 (12)	−0.0065 (13)
C10	0.0353 (15)	0.0589 (17)	0.0338 (15)	−0.0024 (13)	−0.0019 (12)	−0.0091 (13)
C11	0.0410 (17)	0.092 (2)	0.059 (2)	0.0014 (17)	−0.0071 (15)	0.0007 (18)
C12	0.0450 (16)	0.0540 (17)	0.0320 (14)	−0.0026 (13)	−0.0015 (12)	0.0045 (12)
C13	0.0360 (15)	0.0489 (16)	0.0350 (15)	−0.0041 (12)	0.0016 (11)	−0.0074 (13)
C14	0.0441 (17)	0.073 (2)	0.0497 (18)	−0.0077 (15)	0.0059 (14)	0.0017 (15)
C15	0.0391 (15)	0.0421 (15)	0.0369 (15)	0.0007 (12)	−0.0028 (12)	−0.0064 (12)
C16	0.0495 (18)	0.0441 (16)	0.0373 (15)	−0.0046 (13)	0.0004 (13)	−0.0020 (12)
C17	0.0403 (16)	0.0495 (16)	0.0459 (16)	−0.0013 (13)	−0.0059 (12)	−0.0063 (13)
C18	0.0406 (15)	0.0393 (15)	0.0379 (15)	−0.0016 (12)	−0.0013 (12)	0.0030 (12)
C19	0.0554 (19)	0.0470 (17)	0.0455 (17)	0.0052 (14)	−0.0052 (14)	−0.0060 (14)
C20	0.064 (2)	0.0540 (18)	0.058 (2)	0.0218 (16)	−0.0026 (16)	−0.0052 (15)
C21	0.053 (2)	0.070 (2)	0.070 (2)	0.0189 (17)	−0.0132 (17)	0.0002 (18)
C22	0.0482 (18)	0.0578 (19)	0.0544 (19)	0.0003 (15)	−0.0136 (14)	−0.0019 (16)
C23	0.0406 (15)	0.0391 (15)	0.0401 (15)	−0.0011 (12)	−0.0001 (12)	0.0027 (12)
C24	0.0423 (17)	0.0480 (17)	0.0457 (18)	−0.0079 (14)	−0.0008 (14)	−0.0030 (14)
C25	0.0549 (19)	0.062 (2)	0.0505 (19)	−0.0042 (16)	0.0085 (15)	0.0024 (15)
C26	0.053 (2)	0.087 (3)	0.068 (2)	0.001 (2)	0.0125 (17)	−0.015 (2)
C27	0.055 (2)	0.082 (3)	0.088 (3)	0.012 (2)	−0.004 (2)	−0.029 (2)
C28	0.075 (3)	0.064 (2)	0.095 (3)	0.011 (2)	−0.028 (2)	0.002 (2)
C29	0.070 (2)	0.068 (2)	0.057 (2)	0.0068 (18)	0.0022 (17)	0.0139 (18)

### Geometric parameters (Å, °)

**Table:**

Fe1—O1	2.412 (2)	C11—H11A	0.9600
Fe1—O1^i^	2.412 (2)	C11—H11B	0.9600
Fe1—O2^i^	2.0400 (18)	C11—H11C	0.9600
Fe1—O2	2.0400 (18)	C12—C13	1.393 (3)
Fe1—N1^i^	2.100 (2)	C12—H12A	0.9300
Fe1—N1	2.100 (2)	C13—C15	1.405 (3)
S2—C18	1.776 (3)	C13—C14	1.505 (4)
S2—C17	1.823 (3)	C14—H14A	0.9600
S3—C7	1.769 (3)	C14—H14B	0.9600
S3—C8	1.818 (3)	C14—H14C	0.9600
N1—C29	1.327 (4)	C15—C16	1.391 (3)
N1—C25	1.338 (3)	C15—C17	1.508 (3)
O1—C1	1.252 (3)	C16—H16A	0.9300
O2—C1	1.268 (3)	C17—H17A	0.9700
O3—C24	1.206 (3)	C17—H17B	0.9700
O4—C24	1.336 (3)	C18—C19	1.395 (4)
O4—H4	0.96 (4)	C18—C23	1.411 (3)
C1—C2	1.496 (4)	C19—C20	1.374 (4)
C2—C3	1.388 (4)	C19—H19A	0.9300
C2—C7	1.412 (4)	C20—C21	1.377 (4)
C3—C4	1.375 (4)	C20—H20A	0.9300
C3—H3A	0.9300	C21—C22	1.367 (4)
C4—C5	1.365 (4)	C21—H21A	0.9300
C4—H4A	0.9300	C22—C23	1.394 (4)
C5—C6	1.375 (4)	C22—H22A	0.9300
C5—H5A	0.9300	C23—C24	1.489 (4)
C6—C7	1.391 (4)	C25—C26	1.357 (4)
C6—H6A	0.9300	C25—H25A	0.9300
C8—C9	1.516 (3)	C26—C27	1.350 (5)
C8—H8A	0.9700	C26—H26A	0.9300
C8—H8B	0.9700	C27—C28	1.370 (5)
C9—C16	1.392 (3)	C27—H27A	0.9300
C9—C10	1.391 (4)	C28—C29	1.386 (5)
C10—C12	1.383 (4)	C28—H28A	0.9300
C10—C11	1.514 (4)	C29—H29A	0.9300
			
O2^i^—Fe1—O2	147.21 (11)	C12—C13—C14	119.8 (2)
O2^i^—Fe1—N1^i^	101.86 (8)	C15—C13—C14	122.2 (2)
O2—Fe1—N1^i^	99.04 (8)	C13—C14—H14A	109.5
O2^i^—Fe1—N1	99.04 (8)	C13—C14—H14B	109.5
O2—Fe1—N1	101.86 (8)	H14A—C14—H14B	109.5
N1^i^—Fe1—N1	100.07 (13)	C13—C14—H14C	109.5
C18—S2—C17	103.62 (12)	H14A—C14—H14C	109.5
C7—S3—C8	103.45 (13)	H14B—C14—H14C	109.5
C29—N1—C25	117.1 (3)	C16—C15—C13	118.5 (2)
C29—N1—Fe1	122.5 (2)	C16—C15—C17	119.2 (2)
C25—N1—Fe1	119.7 (2)	C13—C15—C17	122.2 (2)
C1—O2—Fe1	98.68 (16)	C15—C16—C9	122.9 (3)
C24—O4—H4	108 (2)	C15—C16—H16A	118.5
O1—C1—O2	120.6 (3)	C9—C16—H16A	118.5
O1—C1—C2	121.0 (2)	C15—C17—S2	108.51 (18)
O2—C1—C2	118.3 (2)	C15—C17—H17A	110.0
C3—C2—C7	119.9 (3)	S2—C17—H17A	110.0
C3—C2—C1	117.7 (2)	C15—C17—H17B	110.0
C7—C2—C1	122.4 (2)	S2—C17—H17B	110.0
C4—C3—C2	121.0 (3)	H17A—C17—H17B	108.4
C4—C3—H3A	119.5	C19—C18—C23	117.5 (2)
C2—C3—H3A	119.5	C19—C18—S2	121.7 (2)
C5—C4—C3	119.3 (3)	C23—C18—S2	120.7 (2)
C5—C4—H4A	120.3	C20—C19—C18	121.4 (3)
C3—C4—H4A	120.3	C20—C19—H19A	119.3
C4—C5—C6	121.0 (3)	C18—C19—H19A	119.3
C4—C5—H5A	119.5	C19—C20—C21	120.9 (3)
C6—C5—H5A	119.5	C19—C20—H20A	119.6
C5—C6—C7	121.3 (3)	C21—C20—H20A	119.6
C5—C6—H6A	119.4	C22—C21—C20	119.1 (3)
C7—C6—H6A	119.4	C22—C21—H21A	120.5
C6—C7—C2	117.5 (2)	C20—C21—H21A	120.5
C6—C7—S3	122.3 (2)	C21—C22—C23	121.5 (3)
C2—C7—S3	120.3 (2)	C21—C22—H22A	119.3
C9—C8—S3	106.88 (18)	C23—C22—H22A	119.3
C9—C8—H8A	110.3	C22—C23—C18	119.7 (2)
S3—C8—H8A	110.3	C22—C23—C24	118.8 (2)
C9—C8—H8B	110.3	C18—C23—C24	121.5 (2)
S3—C8—H8B	110.3	O3—C24—O4	122.7 (3)
H8A—C8—H8B	108.6	O3—C24—C23	124.1 (2)
C16—C9—C10	118.3 (2)	O4—C24—C23	113.2 (3)
C16—C9—C8	119.3 (3)	N1—C25—C26	122.9 (3)
C10—C9—C8	122.4 (2)	N1—C25—H25A	118.5
C12—C10—C9	119.0 (2)	C26—C25—H25A	118.5
C12—C10—C11	119.5 (3)	C27—C26—C25	119.8 (3)
C9—C10—C11	121.6 (3)	C27—C26—H26A	120.1
C10—C11—H11A	109.5	C25—C26—H26A	120.1
C10—C11—H11B	109.5	C26—C27—C28	119.0 (3)
H11A—C11—H11B	109.5	C26—C27—H27A	120.5
C10—C11—H11C	109.5	C28—C27—H27A	120.5
H11A—C11—H11C	109.5	C27—C28—C29	118.2 (3)
H11B—C11—H11C	109.5	C27—C28—H28A	120.9
C10—C12—C13	123.1 (3)	C29—C28—H28A	120.9
C10—C12—H12A	118.4	N1—C29—C28	122.9 (3)
C13—C12—H12A	118.4	N1—C29—H29A	118.5
C12—C13—C15	118.0 (2)	C28—C29—H29A	118.5
			
O2^i^—Fe1—N1—C29	−146.8 (2)	C10—C12—C13—C14	−177.2 (2)
O2—Fe1—N1—C29	58.7 (2)	C12—C13—C15—C16	−2.1 (4)
N1^i^—Fe1—N1—C29	−42.9 (2)	C14—C13—C15—C16	176.3 (2)
O2^i^—Fe1—N1—C25	43.1 (2)	C12—C13—C15—C17	179.6 (2)
O2—Fe1—N1—C25	−111.5 (2)	C14—C13—C15—C17	−1.9 (4)
N1^i^—Fe1—N1—C25	147.0 (2)	C13—C15—C16—C9	−0.4 (4)
O2^i^—Fe1—O2—C1	−39.76 (14)	C17—C15—C16—C9	177.9 (2)
N1^i^—Fe1—O2—C1	−168.90 (15)	C10—C9—C16—C15	3.8 (4)
N1—Fe1—O2—C1	88.73 (16)	C8—C9—C16—C15	−176.1 (2)
Fe1—O2—C1—O1	−7.3 (3)	C16—C15—C17—S2	98.4 (2)
Fe1—O2—C1—C2	174.59 (18)	C13—C15—C17—S2	−83.3 (3)
O1—C1—C2—C3	−16.7 (4)	C18—S2—C17—C15	179.62 (18)
O2—C1—C2—C3	161.4 (2)	C17—S2—C18—C19	−1.8 (3)
O1—C1—C2—C7	163.4 (2)	C17—S2—C18—C23	176.1 (2)
O2—C1—C2—C7	−18.5 (4)	C23—C18—C19—C20	1.8 (4)
C7—C2—C3—C4	0.6 (4)	S2—C18—C19—C20	179.7 (2)
C1—C2—C3—C4	−179.4 (3)	C18—C19—C20—C21	0.7 (5)
C2—C3—C4—C5	−0.8 (5)	C19—C20—C21—C22	−2.0 (5)
C3—C4—C5—C6	0.1 (6)	C20—C21—C22—C23	0.9 (5)
C4—C5—C6—C7	0.9 (6)	C21—C22—C23—C18	1.6 (4)
C5—C6—C7—C2	−1.2 (4)	C21—C22—C23—C24	−177.2 (3)
C5—C6—C7—S3	179.7 (3)	C19—C18—C23—C22	−2.9 (4)
C3—C2—C7—C6	0.4 (4)	S2—C18—C23—C22	179.2 (2)
C1—C2—C7—C6	−179.6 (2)	C19—C18—C23—C24	175.8 (2)
C3—C2—C7—S3	179.5 (2)	S2—C18—C23—C24	−2.1 (3)
C1—C2—C7—S3	−0.5 (3)	C22—C23—C24—O3	−163.3 (3)
C8—S3—C7—C6	19.2 (3)	C18—C23—C24—O3	18.0 (4)
C8—S3—C7—C2	−159.9 (2)	C22—C23—C24—O4	17.1 (4)
C7—S3—C8—C9	177.75 (19)	C18—C23—C24—O4	−161.6 (2)
S3—C8—C9—C16	−82.8 (3)	C29—N1—C25—C26	−2.6 (4)
S3—C8—C9—C10	97.3 (3)	Fe1—N1—C25—C26	168.0 (2)
C16—C9—C10—C12	−4.5 (4)	N1—C25—C26—C27	1.2 (5)
C8—C9—C10—C12	175.4 (2)	C25—C26—C27—C28	0.9 (5)
C16—C9—C10—C11	176.8 (2)	C26—C27—C28—C29	−1.6 (5)
C8—C9—C10—C11	−3.2 (4)	C25—N1—C29—C28	1.9 (5)
C9—C10—C12—C13	2.1 (4)	Fe1—N1—C29—C28	−168.5 (3)
C11—C10—C12—C13	−179.2 (3)	C27—C28—C29—N1	0.2 (5)
C10—C12—C13—C15	1.3 (4)		

Symmetry codes: (i) −*x*, *y*, −*z*+1/2.

### Hydrogen-bond geometry (Å, °)

**Table:**

*D*—H···*A*	*D*—H	H···*A*	*D*···*A*	*D*—H···*A*
O4—H4···O1^ii^	0.96 (4)	1.77 (4)	2.702 (3)	162 (4)
C16—H16A···O3^iii^	0.93	2.54	3.421 (3)	158

Symmetry codes: (ii) *x*−1/2, *y*−1/2, −*z*+1/2; (iii) −*x*−1/2, *y*+1/2, *z*.
